# The shifted balance of arginine metabolites in acute myocardial infarction patients and its clinical relevance

**DOI:** 10.1038/s41598-020-80230-3

**Published:** 2021-01-08

**Authors:** Patrycja Molek, Pawel Zmudzki, Aleksandra Wlodarczyk, Jadwiga Nessler, Jaroslaw Zalewski

**Affiliations:** 1 Department of Coronary Disease and Heart Failure, Jagiellonian University Medical College, 80 Pradnicka Street, 31-202 Krakow, Poland; 2 Faculty of Pharmacy, Jagiellonian University Medical College, Krakow, Poland

**Keywords:** Biomarkers, Cardiology, Pathogenesis

## Abstract

The arginine metabolism as a target for cardioprotection in patients with ST-segment elevation myocardial infarction (STEMI) remains insufficiently understood. Arginine, ornithine, citrulline, asymmetric dimethylarginine (ADMA) and proline plasma levels were measured using liquid chromatography and tandem mass spectrometry in 70 consecutive STEMI patients upon admission and at 6-month follow-up and were compared with left ventricular function, volumes, and infarct characteristics determined by cardiac magnetic resonance imaging, and with 5-year clinical outcomes. Baseline median concentration of arginine was higher by 49% (*P* = 0.002) when compared to 6-month measurements and was correlated with an ischemia risk area (R = 0.34, *P* = 0.004) and infarct size (R = 0.33, *P* = 0.006). Following ischemia median citrulline/arginine index decreased when compared with 6-month result (*P* = 0.002), while citrulline/ornithine and arginine/ADMA ratios maintained unchanged indicating a shift of arginine metabolism from nitric oxide synthase (NOS) towards arginase. The 6-month arginine concentration reached the area under the ROC curve of 0.67 (95% confidence interval 0.54–0.81) for prediction of death, myocardial infarction or heart failure hospitalization and its value of < 29 µM was associated with lower event free survival (*P* = 0.02). In STEMI patients, during ischemia conversion of elevated plasma arginine was shifted from NOS towards arginase. Decreased 6-month arginine concentrations were associated with worse long-term outcomes.

## Introduction

Arginine is both a substrate for arginase and the sole source for nitric oxide (NO) synthesis by three main isoforms of NO synthases (NOS)^1–4^. Competing of both enzymes for the same L-arginine may lead to diminished production of an unstable molecule of NO which undergoes non-enzymatic oxidation to nitrite and finally to stable nitrate. In stable conditions endothelial NOS produces majority of NO, however changes in NO and L-citrulline levels may also reflect activation of inducible NOS especially in response to hypoxia or cardiac injury and non-enzymatic NO formation^5^. It has been demonstrated that NO synthesis is not exclusively attributed to the vascular endothelium but also red blood cells express a functional endothelial NOS and constitutively produce NO^6,7^ and contribute to the regulation of systemic blood pressure and nitrite homeostasis^8^. Apart from NOS and arginase, arginine is a substrate for arginine decarboxylase that synthesize L-agmatine linked directly to polyamine metabolism or for arginine:glycine amidinotransferase producing ornithine, guanidinoacetate and L-homoarginine^9^. In turn, asymmetric dimethylarginine (ADMA) is derived from the methylation of arginine residues of various proteins and acts as an endogenous NOS inhibitor.

There is evidence that endothelial damage following ischemia/reperfusion (I/R) injury is a component of no-reflow phenomenon and favors infarct area expansion^10^. Kloner et al. showed that structural changes in coronary microcirculation were preceded by and limited to the areas of irreversible cardiomyocyte injury^11^, and were substrate for intramyocardial hemorrhage^12^. The endothelial damage is associated with impaired endothelial-dependent vasodilatation and diminished NO bioavailability^13^. In cultured endothelial cells, exposure to hypoxia leads to an increased intracellular concentration of L-arginine released from cellular storages or protein proteolysis^14^ whereas impaired uptake of L-arginine is a consequence of plasma membrane depolarization^15^. Although intra- and extracellular arginine concentration was elevated during I/R, it was inaccessible for NO production due to endothelial damage.

Diminished arginine bioavailability and its dysfunctional metabolism have been shown to be associated with increased cardiovascular risk. Reduced global arginine bioavailability ratio (GABR), but not arginine concentration, was associated with both higher prevalence of coronary artery disease (CAD) and an increased incidence of long-term cardiovascular events^16–18^. Moreover, patients who presented symptoms of cardiogenic shock following MI had plasma levels of arginine that were twice as low, along with a 42% lower GABR when compared with stable CAD patients^19^. Recent studies on red blood cells provide evidence that their endothelial NOS activity is compromised in patients with coronary artery disease^7^ while elevated arginine I activity in patients with type 2 diabetes is associated with induction of endothelial dysfunction^20^.

In animal model of myocardial infarction (MI) a systemic inhibition of arginase was associated with an increase of plasma citrulline, citrulline/arginine ratio and citrulline/ornithine ratio and a decrease of plasma ornithine^21^. Simultaneous assessment using two ratios of citrulline/ornithine and citrulline/arginine provided indirect insight pertaining to the balancing activity of NOS and arginase. In turn, an arginine/ADMA ratio provided information about arginine availability for NO production dependent on NOS inhibitor^22^ and was identified as a marker of heart failure^23^, hypertension^24^, atherosclerosis^25^ and of major cardiovascular events^26^. In contrast, a nitrate anion as a dietary element that can be reduced via nitrite into NO does not reflect NO availability^27^. Lauer et al.^28^ have shown that plasma nitrite, but not nitrate correlated with NO production from endothelial NOS in humans. Additionally, in patients with increased cardiovascular risk, there is a decreased availability of tetrahydrobiopterin which results in an endothelial NOS-dependent formation of superoxide instead of NO and promotes the oxidation of NO to nitrate^29^.

To date an unclear role of arginine metabolism in acute MI precluded the development of effective arginine-based cardioprotective therapies. We hypothesize that in patients with ST-segment elevation myocardial infarction (STEMI) elevated plasma arginine together with its metabolites released following ischemia from damaged cardiac myocytes may reflect myocardial and microvascular injury, a shifted metabolic balance between NOS and arginase and potentially might be useful in prediction of clinical outcomes.

## Methods

### Patients

Seventy consecutive STEMI patients who underwent primary percutaneous coronary intervention (PCI) were enrolled. They qualified for inclusion if presented with chest pain duration at rest no longer than 12 h with concomitant ST-segment elevation > 1 mm in 2 or more limb leads or > 2 mm in 2 precordial leads, or if revealed a new left bundle branch block. Criteria for exclusion included lack of informed consent, the presence of cardiogenic shock or pulmonary edema, history of MI or PCI at the same location or coronary artery bypass surgery, history of cancer, venous thromboembolism, renal or liver failure, and contraindications of cardiac magnetic resonance (CMR) imaging.

The demographic characteristics, anthropometric parameters, cardiovascular risk factors, history of cardiovascular diseases and comorbidities were collected for all patients. Upon admission, hemoglobin, hematocrit, red and white blood cell count, platelet count, lipid profile, glucose, creatinine, high sensitivity C-reactive protein and fibrinogen were determined using standard laboratory techniques. Serum activity of creatine kinase (CK, IU/L), isoenzyme MB of creatine kinase (CK-MB, IU/L) as well as concentration of cardiac high sensitivity troponin T (ng/ml) were measured upon admission and then subsequently at 8, 16, 24 and 48 h. Diagnosis of diabetes mellitus was derived from patient history. A new diabetes mellitus diagnosis was given based upon hyperglycemic presentation at intake or after a glucose tolerance test. Chronic kidney disease was diagnosed when creatinine clearance using the Cockcroft-Gault formula was calculated to be lower than 60 ml/min.

The study was approved by the Ethics Committee of Jagiellonian University and all methods were performed in accordance with the relevant guidelines and regulations. All study participants provided written informed consent prior to their inclusion to the study procedures.

### Quantification of arginine metabolites

Blood samples were drawn into citrate tubes upon admission in acute phase of MI before PCI and again, 6 months later. Before 6-month follow-up visit all patients were on low-nitrate/nitrite diet for three days. The samples were centrifuged at 2,500 g at 18 °C to 22 °C for 20 min and processed immediately or stored in aliquots at − 80 °C until analysis. Plasma proteins for amino acid analysis were precipitated with 80% methanol before measurements were recorded. Plasma concentrations of arginine, ornithine, citrulline, proline, ADMA and nitrite/nitrate (NO_x_) were measured in duplicates using ultra-performance liquid chromatography—tandem mass spectrometer (UPLC-MS/MS) with Waters ACQUITY UPLC (Waters Corporation, Milford, MA, USA) coupled to a Waters TQD mass spectrometer (electrospray ionization mode ESI-tandem quadrupole).

NO_x_ measurement procedure was performed in two ways^30^. For nitrites the procedure included direct derivatization to azo compound—4-((4-((2-aminoethyl)amino)naphthalen-1-yl)diazenyl)benzenesulfonamide, which was later quantified using the developed UPLC-MS/MS procedure. The procedure for nitrates included two stages of enzymatic reduction to nitrites and subsequent derivatization to azo compound and quantification as above described. Procedures in plasma samples were performed in parallel, and the final content of nitrates was calculated based upon the difference between values obtained from both procedures since the second procedure simultaneously quantifying nitrites and nitrates within the sample. Chromatographic separations were carried out using Acquity UPLC BEH C_18_ column, 2.1 × 100 mm, and 1.7 µm particle size equipped with Acquity UPLC BEH C_18_ VanGuard pre-column. The column was maintained at 40 °C and eluted under the following conditions: linear gradient elution from 100 to 20% of eluent A over a 4 min timeframe, followed by isocratic elution from 20% of eluent A over 2 min, and linear gradient elution from 20 to 100% of eluent A over 1 min at a flow rate of 0.3 mL/min (Eluent A: water/formic acid (0.1%, v/v); eluent B: acetonitrile/formic acid (0.1%, v/v). 10 μL of each sample was injected in triplicate.

Chromatographic separations of amino acids were carried out with the Acquity UPLC BEH Amide column, 2.1 × 100 mm, and the 1.7 µm particle size equipped with VanGuard Acquity UPLC BEH Amide pre-column. The column was maintained at 40 °C and eluted under linear gradient elution from 20 to 35% of eluent A over 4 min followed by linear gradient elution from 35 to 40% of eluent A over 6 min, with a flow rate of 0.3 mL/min. (Eluent A: water/formic acid (0.1%, v/v); eluent B: acetonitrile/formic acid (0.1%, v/v)).

Waters’ TQD mass spectrometer parameters were optimized for quantitative analysis using the solutions of L-arginine, N^G^, N^G^-dimethylarginine, L-citrulline, L-ornithine, L-proline, 4-((4-((2-aminoethyl)amino)naphthalen-1-yl)diazenyl)benzenesulfonamide, and chloramphenicol in concentration 10 μg/mL at flow 20 μL/min and mixture of eluent A and B 1:1 (v/v) at flow 0.28 mL/min.

### Coronary angiography

Coronary angiograms were acquired with Axiom Artis dFC (Siemens, Erlangen, Germany) and were analyzed offline using two contralateral projections for each artery before and after angioplasty. All coronary segments were evaluated for the presence of visible thrombus in the epicardial artery, distal embolization during and after primary PCI as well as degree of stenosis based on visual inspection. Epicardial segments with coronary lesions with at least 50% narrowing were considered significant. Epicardial blood flow in the infarct-related artery (IRA) was evaluated using the TIMI scale^31,32^.

### Left ventricular volumes, function and infarct characteristics

CMR imaging was performed using a 1.5-T scanner (Magnetom Sonata Maestro Class, Siemens, Erlangen, Germany) at baseline, between the 2nd and the 4th day following primary PCI, and then again, at 6 months, as previously described^33^. Briefly, using ECG gating technique, left ventricular (LV) function was assessed with cine imaging in a spoiled gradient echo sequence. Following cine sequences, imaging of myocardial edema reflecting area at risk (AAR) of ischemia was performed using the breathhold, T2-weighted sequence. Then, the late-enhancement imaging was performed 10 to 20 min after contrast injection of 0.1 mmol/kg of gadobutrolum (Gadovist, Bayer Pharma AG, Germany) with a gradient echo readout. The entire left ventricle was covered by contiguous, short-axis oriented 8-mm slices in all CMR techniques.

An in-house-developed, vendor-independent, cardiac-dedicated software (CardioViewer, baseline version, Catholic University Leuven, Leuven, Belgium)^34^ was used for blinded analysis of all images. The borders of endocardium and epicardium were delineated on images with the largest and smallest LV volumes in order to determine end-diastolic and end-systolic LV volumes, respectively. Based on LV volumes measurements, LV ejection fraction and myocardial volumes were calculated. The myocardial edema, infarct size (IS), and microvascular obstruction (MVO) were delineated on T2-weigted as well as late gadolinium-enhanced images, respectively. The area of delineation multiplied by the thickness of the slice and by the myocardial tissue density (1.05 g/ml) served as formula to calculate the mass of AAR, IS and MVO. The AAR was expressed as the ratio of LV mass. The hyper-enhanced area of the infarct was expressed using a ratio of myocardial edema or LV mass, while MVO was expressed using a ratio of the infarct area.

### Long-term clinical follow-up

Death, recurrent MI confirmed by enzyme release, stroke, or hospitalization due to heart failure (HF) decompensation were collected within 5-year follow-up at the outpatient clinic. Data was supplemented by an in-person or telephone interview with the patient or in the case of death, data was obtained through an immediate relative. Ischemic symptoms were assessed according to the Canadian Cardiovascular Society (CCS) scale. The HF hospitalization was defined as an episode of cardiac decompensation, including pulmonary edema or worsening of dyspnea up to at least class III-IV according to the New York Heart Association (NYHA) classification requiring hospital admission.

### Statistical analysis

To demonstrate a 10% relative decrease of arginine concentration during 6-month follow-up with a 90% power and a standard deviation of 20% using a P value of 0.05, 67 patients were required in the whole group.

Statistical analyses were performed with SPSS Statistics software (Version 25.0.0.2, IBM, USA) as previously^33^. Categorical variables are shown as numbers (percentages) and compared by Fisher’s exact test. Continuous variables are shown as median (interquartile range). Continuous variables with normal distribution were compared by Student t test whereas non-normally distributed with the Mann–Whitney U test. Between two time-points normally distributed variables were compared with t test for paired samples or in case of non-normal distribution with the Wilcoxon signed-rank. The correlation of two normally distributed variables was calculated with the Pearson test whereas for non-normal distributed data Spearman rank correlation coefficient was performed. Receiver operating characteristic (ROC) curves and the Youden index were used to determine the optimal cut-off value of arginine or NO_x_ and their sensitivity and specificity in prediction of death, recurrent MI or HF requiring hospitalization. A log-rank test was computed to evaluate differences in survival free of death, recurrent MI or HF requiring hospitalization between patients with estimated cut-off values of arginine or NO_x_ concentration. Finally, all independent variables with the potential for confounding both the outcome and exposure were included in the Cox proportional hazard regression to find predictors of long-term death, recurrent MI or HF requiring hospitalization. A two-tailed P value of less than 0.05 was statistically significant.

## Results

Baseline clinical and laboratory characteristics are reported in Table 1. The studied cohort consisted mostly of middle-aged men. About one-sixth of patients were diabetic and half were hypertensive or active smokers. At baseline, the majority of patients were in Killip class 1 and three-fourths had an anterior STEMI. The median time of ischemia was 220 (interquartile range 160 to 350) minutes. At initial contrast injections, occluded IRA with TIMI 0 or 1 flow was found in 63 (90%) patients whereas after the procedure TIMI 2 or 3 flow was restored in 69 (98.6%) patients. A collateral blood flow was scored from 0 to 3 according to the Rentrop classification and was detected in 47 (67.1%), 14 (20%), 7 (10%) and 2 (2.9%) of subjects, respectively. An aspiration thrombectomy was used in 56 (80%) patients and the procedure was completed in all cases using drug eluting stent implantation. Clinical management and medical treatment, including antithrombotic therapy, were accomplished according to the most recent guidelines.

**Table 1:** Baseline characteristics.

	All patients, n = 70
Age	59.0 (50.8, 64.3)
Male gender	54 (77)
Body mass index, kg/m^2^	26.3 (24.5, 29.0)
Anterior wall myocardial infarction	52 (74)
Killip class > 1 on admission	6 (8.5)
**Cardiovascular risk factors**	
Hypertension	35 (50)
Diabetes mellitus	11 (16)
Dyslipidemia	44 (63)
Current smoking	40 (57)
Family history of coronary artery disease	13 (19)
Chronic kidney disease	0
**Comorbidities**	
Prior myocardial infarction	2 (3)
Prior percutaneous coronary intervention	0
History of stroke	0
History of peripheral vascular disease	1 (1.4)
Pre-infarction angina	10 (14)
**Laboratory results upon admission**	
Hemoglobin, g/dL	14.6 (13.7, 15.4)
Hematocrit, %	42.6 (40.3, 45.8)
Platelets, × 10^3^/µL	218 (187, 260)
White blood cells, × 10^3^/µL	11.5 (9.6, 13.9)
Glucose, mmol/L	8,1 (7.0, 9.5)
Creatinine, µmol/L	92.0 (80.5, 105.5)
High sensitivity C-reactive protein, mg/dL	1.50 (1.12, 4.09)
Fibrinogen, mg/dL	3.31 (3.03, 3.88)
Total cholesterol, mmol/L	5.48 (4.91, 6.36)
LDL cholesterol, mmol/L	3.39 (2.75, 4.15)
HDL cholesterol, mmol/L	1.07 (1.02, 1.28)
Triglycerides, mmol/L	1.01 (0.9, 2.02)
Troponin T, ng/mL	0.041 (0.014, 0,147)
Isoenzyme MB of creatine kinase, IU/L	20 (15, 34)
Area under the curve of isoenzyme MB of creatine kinase release within 48 h of myocardial infarction, IU/L x h	3525 (2222, 5625)
**Antithrombotic treatment before admission**	
Aspirin, loading dose of 300 mg p.o	67 (96)
Clopidogrel, loading dose of 600 mg p.o	44 (62.9)
Unfractionated heparin, loading dose of 5000 IU i.v	39 (56)
**Treatment at discharge**	
Aspirin	70 (100)
Clopidogrel	55 (78.6)
Ticagrelor	15 (21.4)
ACEI or ARB	66 (94)
Beta-blocker	65 (93)
Statin	70 (100)

Data are shown as median (interquartile range) or number (percentage).

*ACEI* angiotensin-converting-enzyme inhibitors, *ARB* angiotensin receptor blockers, *LDL* low-density lipoprotein, *HDL* high-density lipoprotein.

### Left ventricular morphology, function and infarct characteristics

The median time interval between STEMI and first CMR was 3 (2–4) days and between the first and second CMR was 6 (5.5–6.5) months. There were no significant differences in LV end-diastolic or end-systolic volumes and LV ejection fraction between baseline and follow-up (Table 2). During follow-up, stroke volume improved (*P* = 0.005) and LV mass decreased (*P* = 0.011).

**Table 2:** Left ventricular volumes, function and infarct characteristics.

	Acute phase of MI	6-month follow-up	*P* value
**Left ventricular volumes and function**			
Left ventricular end-diastolic volume, ml	169 (146, 198)	179 (148, 227)	0.11
Left ventricular end-diastolic volume index, ml/m^2^	89 (78, 103)	94 (80, 114)	0.14
Left ventricular end-systolic volume, ml	96 (78,120)	95 (64, 141)	0.82
Left ventricular end-systolic volume index, ml/m^2^	51.5 (41, 64)	50 (35, 74)	0.89
Stroke volume, ml	73 (59, 84)	81 (70, 82)	0.005
Stroke volume index, ml/m^2^	39 (30,45)	42 (38, 48)	0.005
Left ventricular ejection fraction, %	42 (36, 48)	44 (35, 54)	0.12
**Infarct characteristics**			
Left ventricular mass, g	144 (125, 172)	134 (105, 151)	0.011
Left ventricular mass index, g/m^2^	75 (68, 86)	70 (57, 78)	0.003
Area at risk, g	73 (49, 883)	NA	
Area at risk/left ventricular mass	0.51 (0.37, 0.60)	NA	
Infarct size, g	47 (24, 68)	31 (13, 45)	< 0.001
Infarct size/area at risk	0.65 (0.51, 0.79)	NA	
Infarct size/left ventricular mass	0.34 (0.19, 0.43)	0.21 (0.11, 0.28)	< 0.001
Present microvascular obstruction	54 (77.1)	NA	
Microvascular obstruction, g	3.45 (0.45, 10.60)	NA	
Microvascular obstruction/infarct size	0.09 (0.02, 0.19)	NA	
Microvascular obstruction/area at risk	0.05 (0.01, 0.13)	NA	

Data are shown as median (interquartile range).

*MI* myocardial infarction, *NA* not applicable.

Median AAR was 51% of left ventricle. The IS expressed as the ratio of LV mass significantly decreased from median baseline value of 0.34 to 0.21 after 6 months (*P* < 0.001). The MVO was detected in 54 of 70 patients, and among these subjects, median MVO extent was 8% of IS (Table 2).

### The shifts in balance of arginine metabolites in acute phase of MI

Baseline median concentration of arginine was significantly higher by 49% (*P* = 0.002), ADMA by 42% (*P* = 0.032) and NO_x_ by 28% (*P* < 0.001) when compared to 6-month follow-up results. However, there were no significant differences between baseline and follow-up concentrations of ornithine, citrulline and proline (Table 3).

**Table 3:** Arginine metabolites and their indices.

	Acute phase of MI	6-month follow-up	*P* value
Arginine, µM	61.5 (39.4, 80.4)	31.5 (23.7, 67.3)	0.002
Citrulline, µM	14.0 (7.1, 20.2)	8.3 (5.8, 25.5)	0.699
Ornithine, µM	69.3 (41.1, 101.9)	50.1 (31.0, 88.3)	0.424
Proline, µM	2.50 (1.75, 3.39)	2.73 (1.78, 3.82)	0.057
ADMA, µM	2.08 (1.34, 2.52)	1.22 (0.76, 2.41)	0.032
NO_x_, µM	8.23 (5.94, 10.95)	5.90 (3.82, 8.05)	< 0.001
**Amino acid and NO**_**x**_** indices**			
GABR	0.79 (0.51, 0.99)	0.62 (0.48, 0.84)	0.015
Ornithine/Arginine	1.10 (0.84, 1.65)	1.35 (0.98, 1.79)	0.110
Citrulline/Ornithine	0.19 (0.16, 0.25)	0.21 (0.15, 0.27)	0.858
Citrulline/Arginine	0.22 (0.17, 0.28)	0.25 (0.20, 0.33)	0.002
Arginine/ADMA	30.6 (25.7, 40.3)	30.6 (21.6, 37.6)	0.317
NO_x_/arginine	0.15 (0.09, 0.27)	0.15 (0.10, 0.24)	0.071
NO_x_/ADMA	4.27 (2.51, 8.19)	3.92 (2.31, 7.44)	0.020

Abbreviations: data are shown as median (interquartile range), ADMA: asymmetric dimethylarginine, GABR: global arginine bioavailability ratio, MI: myocardial infarction, NO_x_: nitrite/nitrate.

There were significant correlations between arginine, ornithine and citrulline both on admission as well as 6 months following MI (Supplementary Table S1). Six months following MI but not in acute phase, we found significant correlations between NO_x_ level versus arginine, ornithine and citrulline.

**Figure 1:**
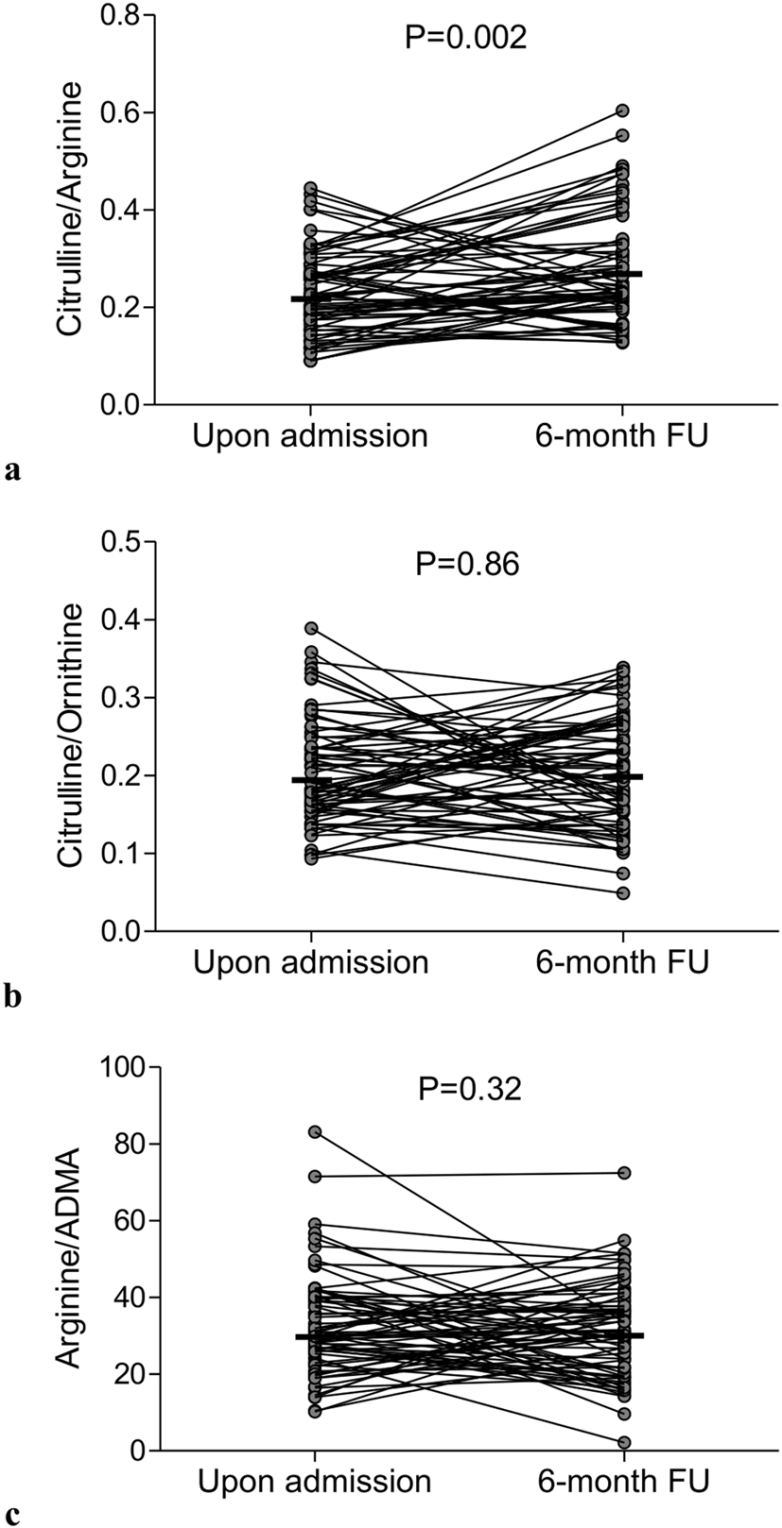
The shifts of balance of arginine metabolites between acute phase of myocardial infarction and stable chronic conditions. Following ischemia (**a**) citrulline/arginine index decreased when compared with stable chronic conditions while (**b**) citrulline/ornithine and (**c**) arginine/ADMA indices maintained unchanged. Data are shown as absolute values, horizontal lines indicate medians. *ADMA* asymmetric dimethylarginine, *FU* follow-up.

There were no significant differences between baseline and follow-up ratios of citrulline/ornithine and arginine/ADMA whereas citrulline/arginine ratio was lower (*P* = 0.002) when compared with 6-month results (Fig. 1).

In acute phase of MI, arginine concentration correlated with fibrinogen and inversely correlated with creatinine and urea (Supplementary Table S2). Simultaneously, ADMA was inversely correlated with creatinine while ornithine and proline were associated with hemoglobin and hematocrit.

### Arginine metabolites and I/R injury

Arginine, ornithine and citrulline upon admission were associated with peak CK-MB release 8 h following PCI (Supplementary Table S3). There was no significant relationship between the concentration of arginine and its metabolites when measured upon admission versus time of ischemia as well as pre- and post-PCI TIMI flow. The concentration of arginine, ornithine, citrulline, proline and ADMA was significantly higher in patients with left anterior descending artery as an IRA compared with the remainder (for all *P* < 0.001). An association was found between collateral blood flow to the IRA classified by Rentrop scale versus arginine (R = 0.26, *P* < 0.05) and NO_x_ (R = 0.25, *P* < 0.05) (Supplementary Fig. S1).

### Arginine metabolites versus CMR findings in acute phase of MI

**Figure 2:**
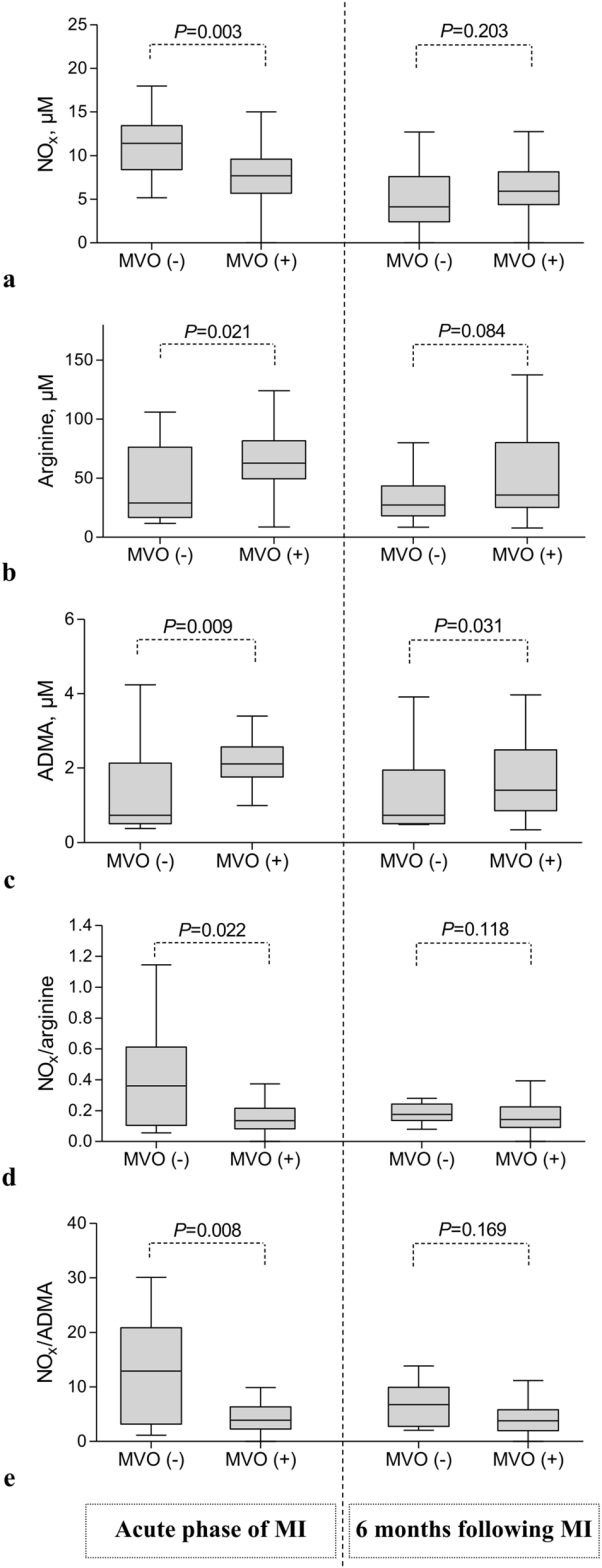
Arginine, ADMA, NO_x_ and their indices in relation to microvascular obstruction. In acute phase of MI, in patients with MVO plasma level of (**a**) NO_x_ was lower but (**b**) arginine and (**c**) ADMA concentrations were higher than in patients without MVO. (**d**) In patients with MVO, NO_x_/arginine and (**e**) NO_x_/ADMA ratios were lower than in subjects without MVO. Apart from ADMA there were no differences between patients with and without MVO at stable chronic phase. Abbreviations: Box plot shows median and interquartile range (IQR, Q3 to Q1). Q1 and Q3 are the first and third quartiles. Whiskers are drawn at minimum and maximum. MVO: microvascular obstruction present ( +) or absent ( −), ADMA: asymmetric dimethylarginine, NO_x_: nitrite/nitrate, MI: myocardial infarction. MVO was determined with an in-house-developed, vendor-independent, cardiac-dedicated software (CardioViewer, baseline version, Catholic University Leuven, Leuven, Belgium) ^34^.

Arginine metabolites measured upon admission in the acute phase of MI were not associated with indexed LV volumes, LV mass or LV ejection fraction as determined with CMR 2–4 days following PCI except for arginine and LV end-systolic volume index (Table 4). However, there were significant correlations between arginine, citrulline, ornithine and ADMA versus AAR expressed as a ratio of LV and IS expressed as a ratio of LV (Table 4). The concentrations of arginine (62.8 [49.6–81.8] versus 29.1 [16.8–76.4] µM, *P* = 0.021) and ADMA (2.11 [1.76–2.57] versus 0.74 [0.51–2.13] µM, *P* = 0.009) were higher but NO_x_ (7.7 [5.7–9.6] versus 11.4 [8.4–13.4] µM, *P* = 0.003) was lower in patients with MVO when compared to subjects without MVO (Fig. 2).

**Table 4:** Arginine metabolites versus cardiac magnetic resonance imaging findings.

	Proline	Citrulline	ADMA	Ornithine	Arginine	NO_x_
**Upon admission during acute phase of myocardial infarction**						
Left ventricular end-diastolic volume index, ml/m^2^						
r	0.052	0.211	0.187	0.171	0.171	− 0.161
P	0.672	0.080	0.122	0.157	0.158	0.184
Left ventricular end-systolic volume index, ml/m^2^						
r	− 0.011	0.192	0.167	0.146	0.238	− 0.040
P	0.931	0.112	0.168	0.229	0.047	0.746
Left ventricular mass index, g/m^2^						
r	0.090	0.081	0.160	0.089	0.165	− 0.101
P	0.459	0.505	0.186	0.465	0.172	0.406
Left ventricular ejection fraction, %						
r	0.075	− 0.095	− 0.082	− 0.054	− 0.234	− 0.113
P	0.536	0.436	0.498	0.657	0.052	0.351
Area at risk/left ventricular mass						
r	**0.253**	**0.371**	**0.342**	**0.359**	**0.337**	− 0.025
P	**0.035**	**0.002**	**0.004**	**0.002**	**0.004**	0.836
Infarct size/left ventricular mass						
r	0.141	**0.254**	**0.327**	**0.268**	**0.365**	− 0.079
P	0.244	**0.034**	**0.006**	**0.025**	**0.002**	0.518
Microvascular obstruction/infarct size						
r	− 0.008	0.091	0.110	0.072	0.186	− 0.049
P	0.948	0.452	0.363	0.555	0.123	0.685
**6-month follow-up**						
Left ventricular end-diastolic volume index, ml/m^2^						
r	**0.242**	− 0.084	− 0.050	0.072	− 0.004	− 0.005
P	**0.045**	0.494	0.685	0.557	0.973	0.970
Left ventricular end-systolic volume index, ml/m^2^						
r	**0.239**	− 0.099	− 0.036	0.070	0.013	0.008
P	**0.047**	0.419	0.770	0.564	0.916	0.951
Left ventricular mass index, g/m^2^						
r	0.252	− 0.068	− 0.025	0.114	− 0.029	0.093
P	0.036	0.581	0.839	0.351	0.811	0.448
Left ventricular ejection fraction, %						
r	− 0.139	0.134	0.026	− 0.051	− 0.014	0.021
P	0.256	0.272	0.834	0.678	0.912	0.865
Infarct size/left ventricular mass						
r	0.163	0.208	0.236	0.212	0.231	0.233
P	0.180	0.086	0.051	0.080	0.060	0.055

*ADMA* asymmetric dimethylarginine, *NO*_*x*_ nitrite/nitrate, arginine metabolites were expressed in µM, *r* correlation coefficient.

### NO_x_ indices versus CMR findings in acute phase of MI

The NO_x_/arginine and NO_x_/ADMA ratios were associated with the burden of ischemia, myocardial injury, and also LV volumes. The lower values of NO_x_/arginine ratio, the bigger indices of AAR/LV and of IS/LV, and the higher indexed end-diastolic and end-systolic LV volumes (Supplementary Table S4). Patients with MVO had lower NO_x_/arginine ratios when compared with subject without MVO (0.13 [0.08–0.22] versus 0.36 [0.11–0.61], *P* = 0.022) (Fig. 2). At baseline, also NO_x_/ADMA ratio was inversely correlated with AAR/LV, IS/LV, LVEDVI and LVESVI (Supplementary Table S4). Patients with MVO had lower NO_x_/ADMA ratio when compared with subject without MVO (3.91 [2.28–6.34] versus 12.90 [3.19–20.84], *P* = 0.007) (Fig. 2).

### Arginine metabolites versus CMR findings in chronic phase

At 6-month follow-up, only proline was correlated with indexed LV volumes and LV mass. Simultaneously, higher values of citrulline/ornithine ratio were associated with smaller LV mass, lower indexed LV volumes, and better LVEF (Table 4).

### Long-term clinical outcomes

All patients completed 1- and 5-year follow-up assessments (Supplementary Table S5). Within the first year, 1 patient died, recurrent MI occurred in 4 survivors, and 2 patients required hospitalization due to HF decompensation. After the first year, 4 additional patients died, a recurrent MI occurred in 6 patients, and 8 patients required hospitalization due to HF. There was no stroke in the studied group. Within 5 years following MI, death, recurrent MI, or HF requiring hospitalization were found in 20 (28.6%) patients. At 5 years, ischemic symptoms with CCS class ≥ 2 were present in 11 patients (20%) and dyspnea with NYHA class ≥ 2 was reported in 19 (34.5%) patients.

**Figure 3:**
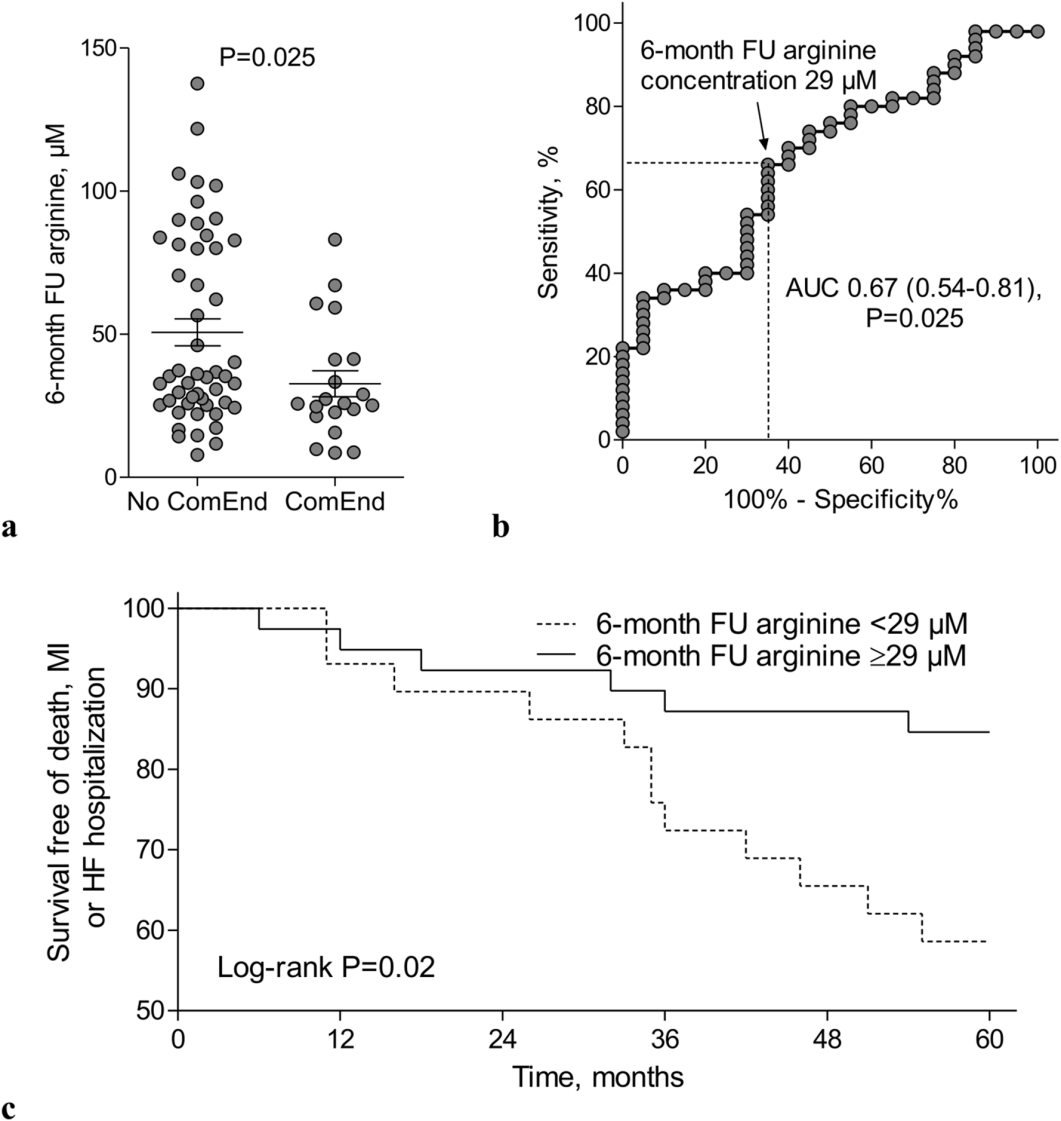
Prognostic value of arginine or NO_x_ concentration. (**a**) The 6-month concentration of arginine was lower in patients with composite endpoint including death, recurrent MI or HF hospitalization during 5-year follow-up. (**b**) At 6-month follow-up arginine concentration reached the area under the ROC curve of 0.67 (*P* = 0.025) for prediction of death, recurrent MI or HF hospitalization with sensitivity of 66% and specificity of 65% and (**c**) its cut-off value of < 29 µM was associated with lower event free survival. *ComEnd* composite endpoint, *HF* heart failure, *MI* myocardial infarction, *NO*_*x*_ nitrite/nitrate, *FU* follow-up, *AUC* area under the receiver operating characteristic curve.

The median time elapsed since baseline blood sampling to UPLC-MS/MS measurements was similar in patients with versus without composite endpoint including death, recurrent MI or HF hospitalization (84 [79–88] versus 84 [80–87] months respectively, *P* = 0.58). There were no correlations between the storage time versus concentrations of arginine metabolites (r from − 0.07 to 0.20, *P* > 0.05 for all) and NO_x_ (r = 0.22, *P* = 0.12). There were no differences in concentrations of baseline arginine (57.5 [24.1–70.8] versus 63.7 [41.1–81.82], *P* = 0.53) between patients with or without composite endpoint. In turn, the follow-up arginine concentration reached the area under the ROC curve of 0.67 (95% confidence interval 0.54–0.81, *P* = 0.025) for prediction of death, recurrent MI or HF hospitalization with sensitivity of 66% and specificity of 65%. Its cut-off value of < 29 µM was associated with lower event free survival (*P* = 0.02) (Fig. 3). By Cox regression, arginine concentration lower than 29 µM when measured 6 months following MI adjusted to age, sex and IS was independently associated with more frequent composite endpoint including death, recurrent MI or HF hospitalization (Table 5).

**Table 5:** The independent predictors of death, recurrent myocardial infarction or hospitalization due to heart failure decompensation.

	Univariable model			Multivariable model		
	*P* value	HR	95% CI for HR	*P* value	HR	95% CI for HR
Age, per 1 year	0.864	1.01	0.97–1.06	0.986	1.00	0.96–1.05
Female gender, yes versus no	0.303	2.17	0.50–9.42	0.386	1.94	0.43–8.71
Infarct size/left ventricular mass, per 0.01	0.550	0.39	0.02–8.56	0.711	0.51	0.02–17.30
Arginine level < 29 µM at 6-month follow-up, yes versus no	0.027	3.02	1.13–8.06	0.047	2.76	1.01–7.57

*CI* confidence interval, *HR* hazard ratio, *NO*_*x*_ nitrite/nitrate.

## Discussion

The present study provides new findings. First, it enables indirect insight into the balance of arginine metabolites in patients with acute MI. Second, the association of arginine metabolites to myocardial I/R injury can strengthen prognostics to improve clinical outcomes.

We have found that plasma levels of ADMA and arginine are elevated following an ischemic phase of MI. Moreover, a higher arginine concentration has been noticed in patients with better developed collateral blood flow to the infarct territory, suggesting their wash out from necrotic myocardium through efficient collaterals. Simultaneously, in acute phase of MI the citrulline/arginine ratio was significantly decreased when compared with stable follow-up phase whereas ratios of citrulline/ornithine and arginine/ADMA maintained unchanged at both time points indirectly indicating that following ischemia conversion of elevated plasma arginine concentration is shifted from NOS towards arginase, despite the lack of enhanced suppressive potential of ADMA for NOS. In turn, the constant balance between metabolites of urea cycle suggested that its enzymes had the same effectiveness in both ischemic phase of MI and in stable chronic conditions, nevertheless it should be remembered that the amount of substrate for arginase was higher in acute condition.

Arginine metabolism in patients with myocardial I/R is insufficiently understood. Most human studies of arginine metabolism examine various pathologies based on indirect methods assessing the concentration of substrates and products involved in arginine-related pathways^16–19,26,35^. Based on the results of animal studies, it is commonly accepted that in cardiac tissue induction of expression and activation of arginase I is an early response to hypoxic stress^36^. At the same time, metabolic activity of NOS competing for the same substrate arginine is reduced or even blocked, because NO formation from arginine requires oxygen^37^. McQuillan et al. proved that during hypoxia expression of constitutive NOS was reduced by 40–60% in the endothelial cells for at least 48 h^38^. The question whether this metabolic shift from NOS towards arginase is beneficial for the survival of ischemic tissues remains a matter of debate. One hypothesis maintains that under the condition of inactive NOS, the reduction of arginine pool by activated arginase may protect cells^36^. The observed shift during acute phase of MI was accompanied by unchanged suppressive potential of ADMA. ADMA however, was identified in several studies as a prognostic marker for cardiovascular risk^39^ including MI patients^40^.

In this study we have shown for the first time that in patients with acute MI, plasma concentration of arginine and its metabolites ornithine, citrulline and ADMA when measured upon admission, were associated with the severity of all morphological features of I/R injury of the left ventricle including AAR, IS and the presence of MVO. Experimental studies indicate that a non-selective NOS inhibitor L-NAME administered 10 min before reperfusion did not reduce no-reflow phenomenon and infarct size^41^ while ischemic preconditioning attenuated both of them via endothelial NOS activation^42^. Recently Fernández-Jiménez et al.^43^ have shown temporal dynamics and extent of edema, IS, MVO and intramyocardial hemorrhage during myocardial I/R. Edema formation had a bimodal temporal distribution and was ameliorated only with cardioprotective interventions. In turn, the area of intramyocardial hemorrhage and MVO varied according to the time of ischemia, CMR timing and cardioprotective strategy. In this context, beyond infarct size reduction also the attenuation of coronary microvascular injury seems to be of particular importance^44–46^. On the other hand, our results indicate that under stable conditions higher proline levels were associated with larger LV volumes and mass. Moreover, the lower the ratio of citrulline/ornithine suggesting metabolic shift from NOS towards arginase, the larger LV volumes and mass. Experimental studies revealed that activation of arginase-dependent polyamine metabolism associated with proline synthesis has a pro-hypertrophic significance^47^.

We found also that the total amount of NO_x_ in stable chronic phase, but not in acute phase of MI, was correlated with plasma concentration of arginine, citrulline and ornithine. Undoubtedly, in acute phase of MI, activation of inducible NOS and the fact that patients were non-fasting could affect NO_x_ plasma level. The concentrations of ADMA and L-arginine as measured by HPLC both in MI patients as well as in healthy volunteers were comparable with our results^48,49^ but NO_x_ levels in healthy volunteers determined with a Griess method were higher than ours^48^. Keeping in mind all the limitations associated with non-fasting measurements of plasma NO_x_ level during acute phase of MI, in this study the new ratios of NO_x_/arginine and NO_x_/ADMA were analyzed. These indices were inversely correlated with both morphological features of I/R injury including territory of ischemia, myocardial necrosis and area of damaged microvasculature as well as with LV end-systolic and end-diastolic volumes. Nevertheless, our preliminary data regarding ratios of NO_x_/arginine and NO_x_/ADMA need to be interpreted with caution, require further validation and their biological relevance remains unknown, the more that NO_x_ cannot be used as a marker of NO production^5,27^.

Can interventions interfering arginine metabolism reduce I/R injury and/or improve MI outcomes? Theoretically, therapies that lead to NO increase may potentially show benefit in cardiovascular disorders, as its decreased concentration plays a key role in multiple dysfunctions. The shifted balance of arginine metabolites from arginase towards NOS found in our study indicates that treatment with arginase inhibitors administered in acute phase of MI may be effective. Jung et al. demonstrated that systemic arginase inhibition reduced IS in rats by 50% in a mechanism dependent on NOS activity and NO bioavailability^21^. Also, local intracoronary arginase inhibitor infusion started 5 min before reperfusion in a pig model of MI reduced IS by 50%^50^. In turn, an intravenous co-administration of L-arginine and tetrahydrobiopterin 5 min before reperfusion reduced IS and this effect was mediated by NOS-dependent pathways resulting in diminished superoxide generation^51^. Although, several specific inhibitors of arginase are available, their systemic mechanism of action is a major limitation of such therapy in humans^1^. Our findings also suggest that in the acute phase of MI, when arginine plasma concentration is high, its supplementation may not bring expected benefit. In contrast, in stable conditions following MI, higher arginine concentrations were associated with better long-term outcomes. Thus, arginine supplementation at that moment might be beneficial. However, available data from VINTAGE MI clinical trial has demonstrated increased mortality associated with arginine supplementation after MI^52^.

Our study has several limitations. First, the analyzed group is relatively small, however sample size was appropriately calculated whereas arginine metabolites and myocardial injury were meticulously measured using the recommended methods. Second, arginine metabolites were not measured in acute phase following reperfusion. Third, non-fasting blood samples in acute phase of MI have been taken, therefore we cannot exclude alternative sources of NO_x_ associated with diet. However, before follow-up blood sampling dietary intake was controlled for nitrate/nitrite. Nevertheless, both acute and chronic measurements of NO_x_ could be confounded by liver metabolism, formation by gastric bacteria, nitrate renal clearance, or contamination^5,27^. Fourth, as the red blood cells are involved in metabolism of arginine and were not examined in this study, the analysis of blood plasma represents only a part of the L-arginine-NO metabolism in the whole blood^6,7^. Another limitation of this study is lack of age matched control group, however all patients were examined twice in acute and chronic phase. Finally, the study population is homogenous in terms of race and ethnicity, thus the data may not be reflective of other more heterogeneous populations.

In conclusion, our findings provide arguments that during acute ischemia in patients with STEMI, conversion of elevated plasma arginine concentration released from damaged cardiac myocytes is shifted from NOS towards arginase, despite the lack of enhanced suppressive potential of asymmetric dimethylarginine for NOS. Measured upon admission, arginine metabolites reflected myocardial and microvascular I/R injury. Simultaneously, decreased arginine concentrations during stable chronic phase were associated with worse long-term clinical outcomes. These findings of potentially clinical relevance may be useful in the development of cardioprotective therapy based on the arginine metabolism in STEMI patients.

## Supplementary Information

Supplementary information.
