# Supplementary material for: The shifted balance of arginine metabolites in acute myocardial infarction patients and its clinical relevance
Source: Sci Rep. 2021 Jan 8;11:83. doi: 10.1038/s41598-020-80230-3 (PMC7794337; doi:10.1038/s41598-020-80230-3)
Supplement: Supplementary file 1 — Supplementary information. [file 41598_2020_80230_MOESM1_ESM.pdf]

Title:
The shifted balance of arginine metabolites in acute myocardial infarction patients and its clinical relevance.

Authors:
Patrycja Molek, Pawel Zmudzki, Aleksandra Wlodarczyk, Jadwiga Nessler, Jaroslaw Zalewski

Supplementary Table S1. Arginine metabolites versus NO_x in acute phase of myocardial infarction and in stable chronic phase.

		Citrulline	ADMA	Ornithine	Arginine	NO_x
Acute phase of MI						
Proline	R	0.600	0.427	0.635	0.556	-0.115
	P	<0.001	<0.001	<0.001	<0.001	0.342
Citrulline	r		0.728	0.842	0.630	-0.099
	P		<0.001	<0.001	<0.001	0.415
ADMA	r			0.759	0.640	-0.158
	P			<0.001	<0.001	0.192
Ornithine	r				0.600	-0.182
	P				<0.001	0.132
Arginine	r					-0.160
	P					0.185
6-month follow-up						
Proline	r	0.487	0.466	0.558	0.402	0.217
	P	<0.001	<0.001	<0.001	<0.001	0.071
Citrulline	r		0.874	0.799	0.694	0.450
	P		<0.001	<0.001	<0.001	<0.001
ADMA	r			0.875	0.715	0.345
	P			<0.001	<0.001	0.003
Ornithine	r				0.604	0.363
	P				<0.001	0.002
Arginine	r					0.330
	P					0.005

Abbreviations: ADMA: asymmetric dimethylarginine, NO_x: nitrite/nitrate, arginine metabolites were expressed in µM, r: correlation coefficient.

Supplementary Table S2. Arginine metabolites and NO_x versus laboratory results upon admission during acute phase of myocardial infarction 6 months later.

		Arginine	Ornithine	Citrulline	Proline	ADMA	NO_x
Hemoglobin, g/dL	r	0.225	**0.315**	0.1746	**0.308**	0.189	**-0.350**
	P	0.061	**0.008**	0.148	**0.010**	0.117	**0.003**
Hematocrit, %	r	0.229	**0.339**	0.198	**0.323**	0.201	**-0.326**
	P	0.057	**0.004**	0.101	**0.006**	0.095	**0.006**
Platelets, $\times 10^3/\mu L$	r	-0.131	-0.118	-0.059	-0.095	-0.002	-0.054
	P	0.279	0.331	0.626	0.433	0.986	0.658
White blood cell count, $\times 10^3/\mu L$	r	-0.092	-0.116	-0.073	-0.232	-0.053	0.200
	P	0.448	0.338	0.549	0.055	0.661	0.096
Glucose, mmol/L	r	-0.095	-0.234	**-0.287**	-0.097	-0.195	0.220
	P	0.440	0.053	**0.017**	0.428	0.109	0.057
Urea, mmol/L	r	**-0.288**	-0.241	-0.144	-0.185	-0.151	0.232
	P	**0.033**	0.076	0.295	0.176	0.270	0.091
Creatinine, μmol/L	r	**-0.353**	-0.236	-0.200	-0.091	**-0.255**	0.174
	P	**0.003**	0.050	0.099	0.455	**0.034**	0.158
High sensitivity C reactive protein, mg/dL	r	-0.023	-0.163	-0.168	-0.187	-0.132	0.005
	P	0.851	0.177	0.163	0.122	0.277	0.966
Fibrinogen, mg/dL	r	**0.313**	0.042	0.120	0.044	0.237	0.093
	P	**0.011**	0.741	0.340	0.731	0.058	0.463
Total cholesterol, mmol/L	r	-0.045	-0.120	-0.168	0.074	-0.028	-0.002
	P	0.720	0.336	0.178	0.557	0.822	0.989
LDL cholesterol, mmol/L	r	-0.123	-0.139	-0.204	-0.038	-0.049	0.032
	P	0.327	0.266	0.100	0.764	0.697	0.797
HDL cholesterol, mmol/L	r	0.116	0.019	0.037	-0.042	0.079	0.006
	P	0.356	0.878	0.769	0.737	0.531	0.964
Triglycerides, mmol/L	r	0.017	-0.034	-0.066	0.172	-0.089	-0.118
	P	0.892	0.792	0.603	0.062	0.484	0.352
Isoenzyme MB of creatine kinase, IU/L	r	0.147	0.062	-0.074	0.058	0.079	-0.173
	P	0.228	0.613	0.548	0.638	0.518	0.156
Troponin T, ng/mL	r	0.039	-0.039	-0.161	-0.040	0.010	-0.017
	P	0.753	0.754	0.190	0.746	0.934	0.893

Abbreviations: ADMA: asymmetric dimethylarginine, NO_x: nitrite/nitrate, LDL: low-density lipoprotein, HDL: high-density lipoprotein, arginine metabolites are expressed in μM, r: correlation coefficient.

Supplementary Table S3. Arginine metabolites and NO_x versus I/R injury.

		Arginine	Ornithine	Citrulline	Proline	ADMA	NO_x
Isoenzyme MB of creatine kinase, IU/L							
8 hours of reperfusion	r	**0.251**	**0.276**	**0.275**	0.164	0.232	-0.096
	P	**0.025**	**0.027**	**0.028**	0.198	0.053	0.453
16 hours of reperfusion	r	0.203	0.088	0.052	0.071	0.225	-0.071
	P	0.111	0.493	0.684	0.579	0.076	0.582
24 hours of reperfusion	r	0.197	0.086	0.043	0.022	0.188	0.042
	P	0.122	0.504	0.738	0.866	0.140	0.747
Troponin T, ng/ml							
8 hours of reperfusion	r	0.179	0.086	0.025	0.004	0.070	0.032
	P	0.159	0.503	0.845	0.976	0.588	0.803
16 hours of reperfusion	r	0.093	-0.013	-0.030	-0.096	0.089	0.042
	P	0.468	0.920	0.812	0.456	0.489	0.742
24 hours of reperfusion	r	0.066	-0.022	-0.007	-0.096	0.071	0.023
	P	0.609	0.863	0.956	0.452	0.583	0.859
TIMI flow in IRA after PCI	r	0.138	0.004	0.009	0.091	0.027	0.093
	P	0.254	0.975	0.940	0.454	0.826	0.444
LAD versus non-LAD as IRA	P	**<0.001**	**<0.001**	**<0.001**	**<0.001**	**<0.001**	0.317

Abbreviations: ADMA: asymmetric dimethylarginine, NO_x: nitrite/nitrate, I/R: ischemia/reperfusion, TIMI: Thrombolysis in Myocardial Infarction, IRA: infarct-related artery, PCI: percutaneous coronary intervention, LAD: left anterior descending, arginine metabolites are expressed in µM, r: correlation coefficient.

Supplementary Table S4. The indices of arginine metabolites and NO_x versus cardiac magnetic resonance imaging findings.

		GABR	Citrulline/ ornithine	Citrulline/ arginine	NO_x/ arginine	NO_x/ ADMA
Upon admission during acute phase of myocardial infarction						
Left ventricular end-diastolic volume index, ml/m^2	r	-0.061	0.028	0.095	**-0.305**	**-0.331**
	P	0.619	0.816	0.437	**0.010**	**0.006**
Left ventricular end-systolic volume index, ml/m^2	r	0.034	0.045	0.045	**-0.314**	**-0.334**
	P	0.779	0.718	0.710	**0.009**	**0.005**
Left ventricular mass index, g/m^2	r	0.051	-0.024	-0.125	-0.115	-0.172
	P	0.677	0.842	0.304	0.350	0.160
Left ventricular ejection fraction, %	r	-0.149	-0.049	0.032	0.216	0.212
	P	0.218	0.685	0.793	0.076	0.083
Area at risk/left ventricular mass	r	-0.079	-0.013	0.119	**-0.465**	**-0.501**
	P	0.515	0.917	0.326	**<0.001**	**<0.001**
Infarct size/left ventricular mass	r	-0.035	-0.043	0.039	**-0.396**	**-0.449**
	P	0.778	0.725	0.749	**<0.001**	**<0.001**
Microvascular obstruction/infarct size	r	-0.002	-0.061	-0.069	-0.208	-0.229
	P	0.989	0.618	0.570	0.089	0.061
6-month follow-up						
Left ventricular end-diastolic volume index, ml/m^2	r	0.113	**-0.313**	-0.122	-0.069	-0.084
	P	0.356	**0.009**	0.326	0.585	0.508
Left ventricular end-systolic volume index, ml/m^2	r	0.078	**-0.326**	-0.145	-0.064	-0.068
	P	**0.523**	**0.006**	0.242	0.616	0.591
Left ventricular mass index, g/m^2	r	**0.024**	**-0.297**	-0.112	-0.044	-0.023
	P	0.842	**0.013**	0.367	0.728	0.854
Left ventricular ejection fraction, %	r	-0.013	**0.342**	0.157	0.044	0.053
	P	0.919	**0.004**	0.204	0.727	0.675
Infarct size/left ventricular mass	r	-0.047	-0.073	0.018	-0.189	-0.220
	P	0.704	0.557	0.888	0.134	0.080

Abbreviations: GABR: global arginine bioavailability ratio, NO_x: nitrite/nitrate, ADMA: asymmetric dimethylarginine, r: correlation coefficient.

Supplementary Table S5. A long-term clinical outcome.

Outcome (n=70)	At 1-year follow-up	At 5-year follow-up
Death	1	5
Myocardial infarction	4	10
Heart failure requiring hospitalization	2	10
Stroke	0	0
Death or myocardial infarction or heart failure requiring hospitalization	7	20
CCS≥2	5	11
NYHA≥2	6	19

Abbreviations: data are shown as numbers, CCS: Canadian cardiovascular Society, NYHA: New York Heart Association

Supplementary Figure S1. Arginine and NO_x in relation to collateral blood flow to the infarct-related artery.

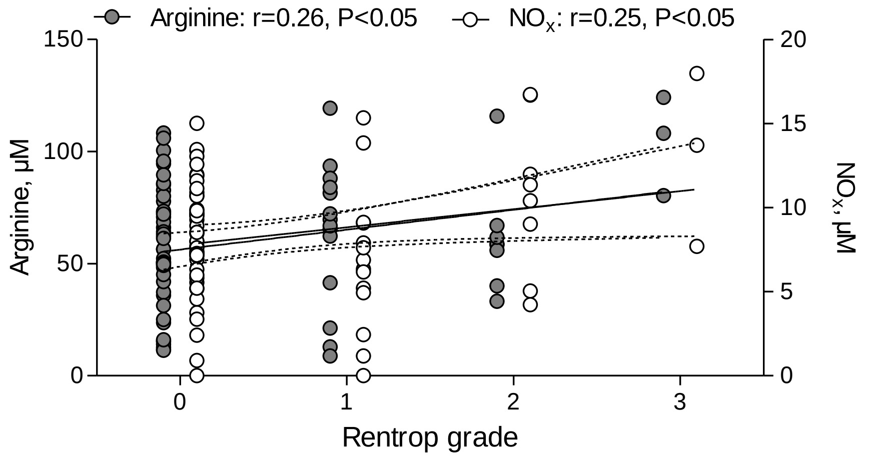

The better collateral blood flow to the infarct-related artery classified by Rentrop scale the higher plasma level of arginine and NO_x.

Abbreviations: data are shown as absolute values. NO_x: nitrite/nitrate, r: correlation coefficient.
